# Keap1/Nrf2 Signaling: A New Player in Thyroid Pathophysiology and Thyroid Cancer

**DOI:** 10.3389/fendo.2019.00510

**Published:** 2019-08-02

**Authors:** Cedric O. Renaud, Panos G. Ziros, Dionysios V. Chartoumpekis, Massimo Bongiovanni, Gerasimos P. Sykiotis

**Affiliations:** ^1^Service of Endocrinology, Diabetology and Metabolism, Lausanne University Hospital and University of Lausanne, Lausanne, Switzerland; ^2^Division of Endocrinology, Department of Internal Medicine, School of Medicine, University of Patras, Patras, Greece; ^3^Service of Clinical Pathology, Institute of Pathology, Lausanne University Hospital and University of Lausanne, Lausanne, Switzerland

**Keywords:** thyroid, Nrf2 (nuclear factor erythroid 2-related factor 2), Keap1 (Kelch-like ECH-associated protein 1), thyroglobulin, oxidative stress, goiter, antioxidant

## Abstract

The Keap1/Nrf2 pathway is a key mediator of general redox and tissue-specific homeostasis. It also exerts a dual role in cancer, by preventing cell transformation of normal cells but promoting aggressiveness, and drug resistance of malignant ones. Although Nrf2 is well-studied in other tissues, its roles in the thyroid gland are only recently emerging. This review focuses on the involvement of Keap1/Nrf2 signaling in thyroid physiology, and pathophysiology in general, and particularly in thyroid cancer. Studies in mice and cultured follicular cells have shown that, under physiological conditions, Nrf2 coordinates antioxidant defenses, directly increases thyroglobulin production and inhibits its iodination. Increased Nrf2 pathway activation has been reported in two independent families with multinodular goiters due to germline loss-of-function mutations in *KEAP1*. Nrf2 pathway activation has also been documented in papillary thyroid carcinoma (PTC), due to somatic mutations, or epigenetic modifications in *KEAP1*, or other pathway components. In PTC, such Nrf2-activating *KEAP1* mutations have been associated with tumor aggressiveness. Furthermore, polymorphisms in the prototypical Nrf2 target genes *NQO1* and *NQO2* have been associated with extra-thyroidal extension and metastasis. More recently, mutations in the Nrf2 pathway have also been found in Hürthle-cell (oncocytic) thyroid carcinoma. Finally, in *in vitro*, and *in vivo* models of poorly-differentiated, and undifferentiated (anaplastic) thyroid carcinoma, Nrf2 activation has been associated with resistance to experimental molecularly-targeted therapy. Thus, Keap1/Nrf2 signaling is involved in both benign and malignant thyroid conditions, where it might serve as a prognostic marker or therapeutic target.

## Introduction

**Figure 1:**
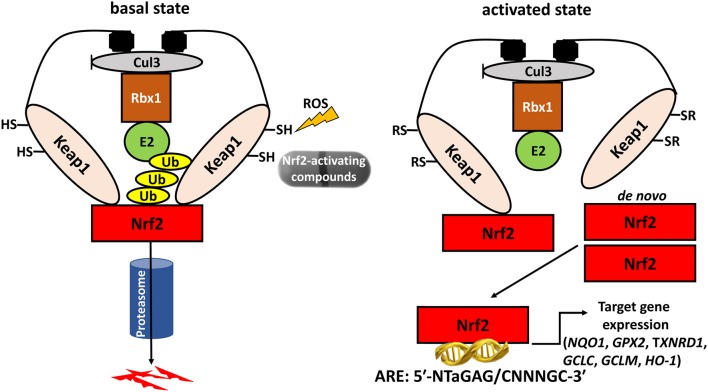
The Keap1/Nrf2 system. Under basal conditions, Nrf2 is bound to its cytoplasmic inhibitory complex formed by Keap1 and Cul3 that targets Nrf2 for polyubiquitination and subsequent degradation by the proteasome. When oxidative stress occurs, the interaction between Nrf2 and the inhibitory complex is abrogated, leading to accumulation of *de novo* synthesized Nrf2 in the nucleus. Nrf2 then promotes the transcription of antioxidant and cytoprotective genes via binding to Antioxidant Response Elements (AREs) located in the genes' regulatory regions. Cul3: Cullin3; Rbx1: RING-box protein 1; E2: ubiquitin-conjugating enzyme 2; Ub: Ubiquitin; Keap1: Kelch-like ECH-associated protein 1; Nrf2: nuclear factor erythroid 2-related transcription factor 2; *NQO1*: NAD(P)H quinone dehydrogenase 1; *GPX2*: glutathione peroxidase 2; *TXNRD1*: thioredoxin reductase 1; *GCLC*: glutamate-cysteine ligase, catalytic subunit; *GCLM*: glutamate-cysteine ligase, modifier subunit; *HO-1*: heme oxygenase 1.

Reactive oxygen species (ROS), such as hydrogen peroxide (H_2_O_2_), are required for normal thyroid cell proliferation as well as for synthesis of the main hormones secreted by thyroid follicular cells, triiodothyronine (T3), and thyroxine (T4) (1–3). However, an unchecked excess of ROS can cause oxidative stress (OS), a factor involved in the pathogenesis of a broad spectrum of diseases, including inflammation, and cancer (4). Thus, thyroid follicular cells need to protect themselves against OS, and recent research has shown that one such protective mechanism is the antioxidant response pathway centered on the nuclear factor erythroid 2-related transcription factor 2 (Nrf2). Nrf2 is a conserved leucine zipper protein that plays a central role in tissue proteostasis by upregulating the transcription of a battery of antioxidant defense genes, and downregulating the transcription of proinflammatory cytokines (5–7). In basal conditions, Nrf2 is bound to its cytoplasmic inhibitory complex formed by Kelch-like ECH-associated protein 1 (Keap1) and Cullin 3 (Cul3), wherein Keap1 targets Nrf2 for polyubiquitination by Cul3 leading to subsequent degradation via the proteasome. Under conditions of OS, specific redox-reactive cysteines of Keap1 become oxidized, thereby abolishing its ability to target Nrf2 for polyubiquitination, and degradation (8–10). Nrf2 is thus stabilized and accumulates in the nucleus, where it binds to DNA sequences called Antioxidant Response Element (AREs) that are located in the promoters, and enhancers of its numerous target genes (11). A model illustrating the pathway, its activation mode, and some of its main target genes is shown in Figure 1. The major importance of Nrf2 in health preservation has been convincingly demonstrated via studies in Nrf2 knockout (KO) mice. Tissues of these mice show decreased expression levels of antioxidant and cytoprotective genes, and proteins like NAD(P)H quinone oxidoreductase 1 (Nqo1, a prototypical Nrf2 target gene), glutathione peroxidase 2 (Gpx2), and thioredoxin reductase 1 (Txnrd1). Conversely, oxidative damage to various tissues is increased (12). Nrf2 KO mice are viable, and fertile (13), but they are highly sensitive to challenges with various factors that trigger OS or other related cellular stresses; as a result of such exposures, Nrf2 KO mice develop respective pathologies, and thus serve as experimental models for the corresponding diseases (12, 14). This multiple-organ protection effect is likely due to the fact that Nrf2 not only regulates a wide range of ubiquitous cell-protective genes, but it also regulates the expression of tissue-specific genes involved in the specialized functional, and homeostatic mechanisms of each respective tissue (15). Nrf2 is ubiquitously expressed and it has been well-studied in several tissues; however, its roles in the thyroid gland are only recently starting to be addressed, with emerging evidence that supports indeed the existence of both general antioxidant as well as thyroid-specific physiological functions (16). This review summarizes the recent work on Keap1/Nrf2 signaling in thyroid physiology, and pathophysiology in general and in thyroid cancer in particular.

## Physiology

Recent studies have shown that Nrf2 is a key antioxidant player in the thyroid gland. *In vivo* work using mice and rats demonstrated that Nrf2 promotes the transcription and protein synthesis of antioxidant and cytoptotective molecules such as Nqo1, Gpx2 and Txnrd1 in the thyroid gland (16, 17); of note, the latter two were long known to have roles that are necessary for the proper functioning of thyroid follicular cells (18, 19). *In vitro* studies in thyroid follicular cells indicate that these regulations take place in a cell-autonomous manner (16). These effects are present in basal conditions (16), and they are much more prominent in conditions of iodine overload (16, 17). Indeed, pharmacological doses of iodine induce the production of oxidative substances in thyroid follicular cells (16, 17). Given the fact that iodine is a fundamental component of thyroid hormones, this potentially reflects an exacerbation of a physiological phenomenon, whereby a certain oxidative state is necessary to facilitate normal thyroid hormone synthesis (2). In wild-type mice, despite an increased oxidative burden in response to iodine overload, oxidized protein and lipid levels do not increase (16); this indicates that endogenous antioxidant defenses are mobilized to prevent OS. Importantly, this protection is lost in Nrf2 KO mice, whose thyroid tissue shows increased levels of oxidized proteins, and lipids in response to pharmacological doses of iodine (16). Indeed, such exposures induce the transcription of genes encoding antioxidant and cytoprotective proteins like Nqo1, and Gpx2 (16, 17); this induction occurs in a Nrf2-dependant manner, because it is abolished in Nrf2 KO mice (16). Thus, it appears that Nrf2 plays a fundamental role in the protection of thyroid follicular cells against the constant oxidative conditions induced by iodine.

Interestingly, activation of Nrf2 by iodine, with subsequent upregulation of its target genes, has also been documented in human skin (20). This suggests that there may exist at least two mechanisms whereby iodine activates Nrf2: one that is specific to the thyroid and is related to the physiological oxidation reactions involving iodine as part of the process of thyroid hormone synthesis; and another that is either specific to the skin or shared among tissues.

**Figure 2:**
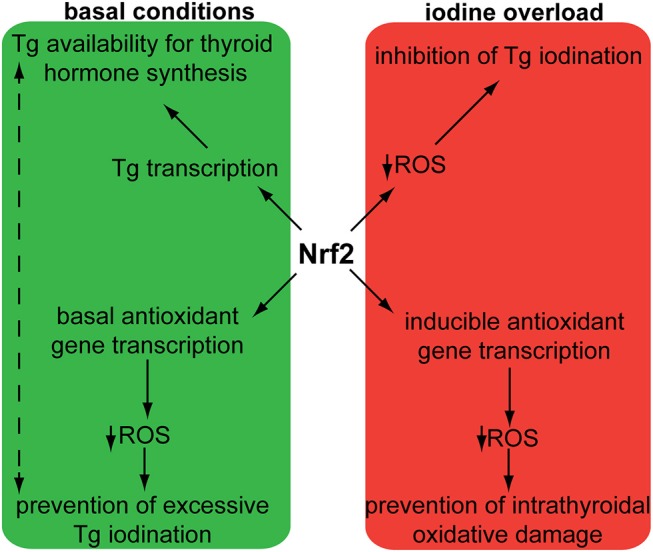
Schematic representation of demonstrated and proposed pleiotropic functions of Nrf2 in follicular thyroid cells under basal conditions and in response to iodine overload. The solid arrows indicate mechanisms that have been sufficiently demonstrated experimentally. Nrf2: nuclear factor erythroid 2-related transcription factor 2; ROS: Reactive Oxygen Species; Tg: thyroglobulin. The dotted arrows indicate putative mechanisms that require further investigation. Originally published in Ziros et al. (16); used here with permission from Mary Ann Liebert Inc.

In addition to its antioxidant defense effects, Nrf2 also plays a specific role in thyroidal functions. Studies in mice and cultured follicular cells have shown that Nrf2 has a dramatic impact on both the basal and the thyroid-stimulating hormone (TSH)-induced intra-thyroidal abundance of thyroglobulin (Tg) (16), which is the main protein produced by the gland, and the precursor molecule of T3 and T4. Nrf2 positively regulates the transcription of the gene encoding Tg via direct binding to two AREs in a conserved upstream enhancer (16). In Nrf2 KO mice, Tg production is effectively reduced; the same is true in cultured follicular thyroid cells, both in basal conditions, and in response to TSH stimulation (16). Another striking effect of Nrf2 is that it decreases Tg iodination, which is an essential step in thyroid hormone synthesis; in Nrf2 KO mice, the thyroidal levels of iodinated Tg are thus highly increased, especially in response to excess iodine (16). The mechanisms involved in this latter phenomenon warrant further elucidation; one proposed hypothesis is that Nrf2 activation reduces the levels of oxidative species, thereby reducing the efficiency of the oxidative reactions involved in Tg iodination. The various effects of Nrf2 on thyroid physiology are summarized in Figure 2.

In summary, Nrf2 emerges as an important regulator of thyroid follicle physiology: it increases the levels of Tg needed for hormone synthesis, prevents its excessive iodination, and protects against intrathyroidal oxidative damage, especially under conditions of iodine overload.

## Goiter

**Figure 3:**
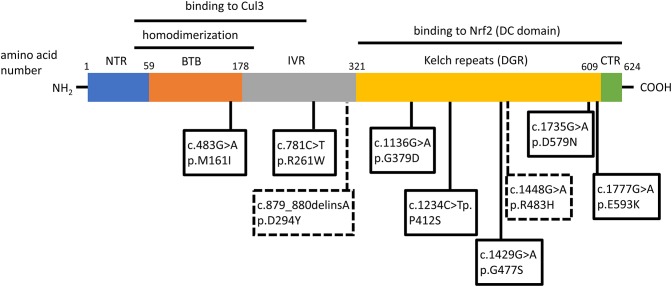
Schematic structure of the Keap1 protein and mutations involved in thyroid gland disorders. Functional regions are indicated with corresponding amino acid number. Dotted lines and rectangles represent germline mutations involved in hereditary multinodular goiter. Solid lines and rectangles represent somatic mutations involved in thyroid cancers. Cul3: Cullin 3; NTR: netrin-like domain; BTB: Broad-Complex, Tramtrack and Bric-a-Brac; IVR: Intervening Region; DRG: Double-Glycine Repeat; DC: DGR and CTR, double-glycine repeat and C-terminal region; CTR: C-terminal Region; NTR: N-terminal Region; NH_2_: amino-terminal end; COOH: carboxy-terminal end.

Two independent case reports have described patients with hereditary multinodular goiters who harbored respective germline loss-of function mutations of *KEAP1*, leading to increased Nrf2 activation (21, 22). The first mutation was found in a 5-generation Japanese family presenting familial non-toxic goiter inherited in an autosomal dominant pattern (21). Genetic analysis revealed a heterozygous mutation in exon 3 of *KEAP1*, resulting in a single base-pair (bp) deletion and frameshift mutation (c.879_880delinsA, p.Asp294Thr, fs^*^23). This mutation affects the IVR (intervening region) domain of Keap1, which is responsible for its dimerization and its interaction with Cul3, as shown in Figure 3. In affected individuals, no Keap1 protein was generated by the mutant allele in the thyroid, and thus the total wild-type Keap1 protein levels in the thyroid were decreased. The mRNA levels of the gene encoding Nrf2 (*NFE2L2*, for NFE2-like 2) were unchanged, but the mRNA levels of *GSTA4* (Glutathione S-transferase A4), and *GCLC* (glutamate cysteine ligase, catalytic subunit) were increased (21). Since both these genes are known to be transcriptionally activated by Nrf2 in other tissues (23, 24), the data indicate that the heterozygous *KEAP1* loss-of-function mutation leads to activation of Nrf2.

The second germline mutation was described in a middle-aged Japanese woman with coexisting non-toxic multinodular goiter and Graves' disease, whose family history was also notable for goiter in her father, and two paternal aunts (22). Genetic analysis identified a heterozygous single point mutation (c.1448G>A, p.R483H) in *KEAP1*, affecting the Keap1 protein's DC (DGR and CTR, double-glycine repeat and C-terminal region) domain, responsible for Nrf2 binding, as shown in Figure 3 (22). Histological analysis of the patient's thyroid nodules after total thyroidectomy showed increased Nrf2 nuclear accumulation, indicating altered Keap1/Nrf2 interaction (22). Interestingly, this same mutation had been previously described as a somatic event in non-small cell lung carcinoma tissue samples, where it was also accompanied by increased Nrf2 nuclear accumulation and by increased mRNA levels of *NFE2L2*, which is itself a target gene of Nrf2 (25), as well as by increased mRNA levels of the Nrf2 target genes *NQO1*, and *MRP2* (multidrug resistance protein 2) (26).

These two independent examples (21, 22) show that loss-of-function mutations in *KEAP1* can be a driver event in some rare forms of hereditary non-toxic multinodular goiter, and suggest that the genetic activation of Nrf2 is a likely goitrogenic mechanism in such cases. The relevance of these observations for the pathogenesis of more common forms of sporadic nodules, and goiters remains to be addressed. However, regardless of any broader relevance for the pathogenesis of goiter, these cases are important because they suggest that there may be some particularities regarding Keap1/Nrf2 signaling in the thyroid as compared to other tissues: It can be reasonably assumed that in the patients harboring germline *KEAP1* loss-of-function mutations, Nrf2 was activated in all tissues. Yet, only the thyroid showed a prominent clinical phenotype leading to the diagnosis, and no extra-thyroidal diseases were reported in the same patients (21, 22). It is thus possible in theory that *KEAP1* haploinsufficiency may lead to more potent Nrf2 activation in the thyroid as compared to other tissues, or that the thyroid may be more sensitive than other tissues to similar increases in the activation status of the Nrf2 pathway (or both). Systematic surveillance, and extended phenotyping of the affected individuals in these families, as well as of any others that may be identified in the future, could help address this very intriguing question.

Another question raised by these observations concerns the thyroidal safety of compounds that activate Nrf2. One such compound, dimethyl fumarate (DMF) is already approved for the treatment of two different diseases (multiple sclerosis and psoriasis), and several others are being tested in clinical trials for other indications (27). Such indications include both the treatment of specific diseases, as well as the prevention of various pathologies associated with OS and/or environmental exposures to toxicants or pollutants, the so-called “chemoprevention” of disease (27). The cases of patients with *KEAP1* loss-of-function mutations described above suggest that the constitutive and life-long genetic activation of Nrf2 can lead to goiter (21, 22). This raises the question of whether the pharmacological activation of Nrf2 in children or adults (potentially lasting from weeks to decades, depending on the disease and the treatment), may have similar effects. It therefore appears prudent to monitor both thyroid function and thyroid volume (at least by palpation) in patients treated with Nrf2-activating compounds on a therapeutic or experimental basis. In that sense, consumption of a broccoli sprout beverage (yielding pharmacologically active amounts of the Nrf2-activating compound sulforaphane) has been recently shown to be safe for thyroid hormonal, and autoimmune status during a 12-week randomized trial (28). Other Nrf2-activating regimens should also be tested on an individual basis, to ensure their safety for the thyroid gland. Priority should be given to antioxidant compounds, known or suspected to affect thyroid function from *in vitro*, and preclinical studies, as summarized in a recent review (29).

## Thyroid Carcinomas

### Pathogenesis and Prognosis

Despite the low rate of proliferation of thyroid follicular cells (30), thyroid tumors (benign or malignant) are quite frequent in the general population. Indeed, even though human thyroid cells divide only about five times during adulthood (30), the spontaneous mutation rate is much higher in the thyroid than in other tissues (31). This might account, at least in part, for the relatively high incidence of thyroid malignancies. About 1.3% of men and women will be diagnosed with thyroid cancer in their lifetime, and this incidence is growing in the last decades (32). Regarding the types of mutations found in thyroid carcinomas, genetic analyses indicate that single-base modifications (but not strand breaks or abasic sites) are more frequent than in cancers of other organs (31). Given that OS preferentially induces single-base mutations (33), these data suggest that mutations found in thyroid carcinomas are mainly due to OS-related DNA damage. In that context, the oxidant H_2_O_2_, a prerequisite for iodine oxidation and Tg iodination, could be causally implicated in the high mutagenesis rate of the thyroid (34), further supporting the importance of antioxidant mechanisms to ensure redox homeostasis. Furthermore, cells of established tumors also try to protect themselves against excessive OS in order to prevent apoptosis (35). These considerations suggest that the Keap1/Nrf2 pathway, as a key regulator of antioxidant defenses, may play important roles in the pathophysiology of thyroid carcinomas. In that sense, it is also noteworthy that Nrf2 has been described to participate in the regulation of DNA repair signaling after induction of DNA damage by ionizing radiation to colonic cells (36). Given that ionizing radiation to the neck before adulthood is a well-known risk factor for thyroid cancer (37), Nrf2 activation could potentially be a means of prevention against carcinogenesis in this case. More generally, based on studies of carcinomas arising in various other tissues, Nrf2 is known to exert a dual role in cancer, by preventing cell transformation of normal cells but promoting aggressiveness, and drug resistance of malignant ones (38–41). The various studies discussed below demonstrate that these concepts extend also to thyroid carcinomas, and they highlight some particularities of the involvement of Keap1/Nrf2 signaling in these specific tumors.

In papillary thyroid carcinoma (PTC), the most common thyroidal malignancy, immunohistochemical analysis showed that the protein levels of Nrf2 and Nqo1 were higher in carcinoma tissues compared to benign follicular adenomas and hyperplastic nodules; both proteins were undetectable in normal thyroid parenchyma adjacent to the PTC samples (42). The oxidized lipid 4-hydroxynonenal (4-HNE) was more abundant in PTC than in adjacent normal tissue, indicating the presence of OS in the cancer, and suggesting that antioxidant defense in PTC is somehow impaired, and/or insufficient to prevent oxidative damage (42). Interestingly, there was no correlation between the protein levels of Nrf2 and Nqo1 in PTC samples, neither between the protein levels of Nrf2 and Keap1; these observations argue against Keap1 downregulation as the principal mechanism of Nrf2 overexpression in PTC, and further suggest that the Nrf2 pathway is not only activated but also at least partially perturbed in PTC (42). In the same study, *in vitro* work with cell lines representing normal human thyrocytes and PTC cells showed that Nrf2 pathway activation promoted the viability of the PTC cell lines but not of normal cells, suggesting that inhibition of Nrf2 may be a potential therapeutic strategy in PTC (42).

In order to characterize the molecular mechanisms leading to Nrf2 activation in thyroid carcinoma, mutations in the Keap1/Nrf2 pathway were sought (42–44). *KEAP1*, and *NFE2L2* somatic mutations have been identified and characterized in various human cancers, including lung, liver, renal, and squamous cell cancers, leukemia, and others (40). Sequencing of PTC samples did not reveal any mutations in *NFE2L2* (and particularly in its known mutational hotspot in exon 2 that encodes one of the domains responsible for the binding to Keap1) (42–44); however, several different mutations were identified in *KEAP1*, albeit at a very low overall frequency (43, 44). The different somatic mutations identified in *KEAP1* in thyroid tumors are shown in Figure 3, Table 1. This is consistent with data showing that, in thyroid tumors overexpressing Nrf2, *NFE2L2* mRNA levels are not increased and may even be decreased, indicating that increased Nrf2 expression is due to post-transcriptional alterations rather than direct transcriptional upregulation (44). In carcinomas harboring a *KEAP1* mutation, immunohistochemical analysis showed increased expression of Nrf2 in comparison with normal parenchyma, suggesting a decreased inhibitory effect of Keap1 on Nrf2 (43).

**Table 1:** List of *KEAP1* mutations involved in benign and malignant thyroid diseases.

**Mutation**	**Inheritance**	**Localization**	**Biological effect**	**Associated disease**
c.879_880delinsA, p.D294Y, fs*23	Germline	IVR domain	Increased Nrf2 pathway activity	familial non-toxic multinodular goiter (NTMG) (21) [also found in somatic form in lung adenocarcinoma (45)]
c.1448G>A, p.R483H	Germline	DC domain	Nrf2 nuclear accumulation	familial NTMG (22) [also found in somatic form in lung squamous-cell carcinoma (26)]
c.483G>A, p.M161I	Somatic	BTB domain	Nrf2 protein overexpression	PTC, tall-cell variant (43)
c.781C>T, p.R261W	Somatic	IVR domain	Nrf2 protein overexpression	PTC, classical variant (43)
c.1136G>A, p.G379D	Somatic	DC domain (Kelch 2)	Reduced Keap1-Nrf2 interaction Nrf2 protein overexpression	PTC, classical variant (43) [also found in somatic form in gallbladder adenocarcinoma (46)]
c.1234C>T, p.P412S	Somatic	DC domain (Kelch 3)	Nrf2 protein overexpression Nrf2 nuclear accumulation	PTC, classical variant (43) [also found in somatic form in ovarian carcinoma (47)]
c.1429G>A, p.G477S	Somatic	DC domain (Kelch 4)	Not tested	PTC, unspecified variant (44)
c.1735G>A, p.D579N	Somatic	DC domain (Kelch 6)	Nrf2 protein overexpression	PTC, follicular variant (43)
c.1777G>A, p.E593K	Somatic	DC domain (Kelch 6)	Nrf2 protein overexpression	PTC, classical variant (43)

*IVR: Intervening Region; DC: DGR and CTR, double-glycine repeat and C-terminal region; BTB: Broad-Complex, Tramtrack and Bric-a-Brac; Nrf2, nuclear factor erythroid 2-related transcription factor 2; PTC, papillary thyroid carcinoma*.

In addition to mutations in the genes encoding the core components of the pathway (*KEAP1* and *NFE2L2*), modifications of their promoter sequences and of genes encoding other regulators of the pathway were also considered (44). Indeed, alterations of genes encoding the regulators comprising the Keap1/Cul3/Rbx1 E3-ubiquitin ligase complex that targets Nrf2 for proteasomal degradation, appear to be extremely frequent in PTC, because more than 80% of samples harbored a DNA alteration in at least one component of this complex (44). In contrast with DNA mutations, which were rare, copy number loss and promoter hypermethylation were often present; for example, these were the most common alterations affecting *RBX1*, and *KEAP1*, respectively. Hypermethylation in the promoter region of genes can cause gene silencing and this phenomenon can contribute to carcinogenesis (48). Silencing of *KEAP1* gene by hypermethylation has been described in several cancers (49). Specifically, *KEAP1* gene hypermethylation is associated with stabilized Nrf2 and increased expression of Nrf2 target genes in lung (45), colorectal (50), and prostate cancer (51). In cancers harboring such epigenetic alterations, prognosis is generally worse because cancer evolution is often more rapid (49).

In PTC samples harboring such genetic alterations, despite reduced levels of *NFE2L2* mRNA, the mRNA levels of Nrf2 target genes were increased, consistent with Nrf2 pathway activation (44). Indeed, genes overexpressed in PTC were enriched in binding sites for the transcription factors c-Jun or Bach1/Bach 2 (44), which are positive and negative regulators, respectively, of Nrf2 signaling (52–55). Taken together, these data indicate that the Nrf2 transcriptional program is activated in PTC due to frequent concerted genetic mechanisms that disrupt multiple components of the Nrf2 inhibitory complex (44).

Mutations in *NFE2L2* itself are very rare in PTC, with only one copy number gain in a single sample reported to date (44). Occasional *NFE2L2* and *KEAP1* mutations were recently identified as part of whole-exome sequencing analysis of patient cohorts with Hürthle-cell (oncocytic) thyroid carcinoma (HCC) (56, 57), a type of differentiated thyroid carcinoma characterized by cells with abundant but dysfunctional mitochondria.

To further characterize Nrf2 pathway activation in thyroid carcinoma, some studies focused on specific upstream regulatory proteins. One such candidate was BRAF, because it is frequently activated by somatic mutation in PTC, and because it is a component of the mitogen-activated protein kinase (MAPK) pathway that is known to activate Nrf2 signaling in various contexts. Among PTC samples with strong expression of Nrf2, the *BRAF* V600E mutation was not more frequent compared to a general PTC cohort, suggesting that Nrf2 activation is not exclusively associated with *BRAF* mutation (42). Another study focused on neuregulin 1 (NRG1), a member of the epidermal growth factor-like family, which was found to be overexpressed in PTC (58, 59). NRG1 positively impacts Nrf2 protein levels in PTC and stimulates the upregulation of its target genes, including *NQO1, GCLC*, and *GCLM* (glutamate cysteine ligase, modulatory subunit) (58). The latter genes improve redox balance, as reflected in a higher ratio of reduced to oxidized glutathione (GSH/GSSG, GSH being the most abundant intracellular antioxidant), and this additional protection against ROS confers a survival advantage to the tumor cells (58). Similar to experimental knock-down of Nrf2 (42), knock-down of NRG1 abolished the survival advantage of PTC cells, suggesting that NRG1 may be a potential therapeutic target (58).

In PTC, some studies have associated the activation of the Keap1/Nrf2 pathway with more aggressive disease. In one such study, patients with the classical variant or tall-cell (more aggressive) variant of PTC whose tumors harbored somatic mutations in *KEAP1*, showed more frequent extra-thyroidal extension, and lymph node metastases (two thirds of cases), and distant metastasis (one third of cases) (43). Moreover, these patients were all classified as being at intermediate or high risk for recurrence according to the criteria established by the American Thyroid Association (60). Of note, *KEAP1* mutations were identified in only a small percentage of patients (<5%) (43). These findings suggest that the identification of somatic *KEAP1* mutations in thyroid carcinomas might serve as an additional prognostic factor. This concept has been previously established in patients with non-small cell lung carcinoma (a tumor with a much higher rate of *KEAP1* somatic mutations), where the presence of such mutations is associated with decreased disease-free survival and decreased overall survival (26).

Papillary thyroid microcarcinoma (PTMC, i.e., PTC of ≤1 cm), is often an indolent disease that does not always warrant surgery but may be eligible for active surveillance (61). However, occasional cases of PTMC show more aggressive behavior, and can even give local or, more rarely, distant metastases. It would thus be very useful to identify factors that could predict aggressive behavior in PTMC, as such patients would not be good candidates for active surveillance. In that sense, germline genetic polymorphisms in *NQO2*, a target gene of Nrf2 (62), were associated with more aggressive behavior of PTMC (63). Specifically, *NQO2* is known to harbor a tri-allelic polymorphism that consists of a 29 bp insertion (I29), a 29 bp deletion (D), and a 16 bp insertion (I16). Patients with PTMC who were homozygous for the *NQO2* I29 allele were more likely to have lymph node metastasis at diagnosis compared with PTMC patients bearing the D allele (63). The association between *NQO2* I29 homozygosity and lymph node metastasis in PTMC was confirmed in multivariate analysis. Of note, the prevalence of the different polymorphisms was similar in patients with PTMC, and patients with benign hyperplastic nodules (63); this indicates that the polymorphism is not implicated in the initiation of PTMC but rather in its progression.

The same study (63) also evaluated a polymorphism in *NQO1*, the C609T missense variant, called NQO1^*^2. Previously, this polymorphism had been associated with higher cancer risk and worse prognosis in breast cancer (64–67), as well as with higher risk for occupational benzene poisoning, which is a risk factor for leukemia (68). In PTMC, the NQO1^*^2 allele was associated with extra-thyroidal extension, but without statistical significance in multivariate analysis (63). It thus does not appear to have the same prognostic value as the *NQO2* I29 allele (63). Regarding Nqo1, in another study there was no correlation between the protein levels of Nqo1 (or Nrf2) with either PTC variants associated with more aggressive behavior (tall-cell, solid/trabecular, diffuse sclerosing, and oncocytic PTC variants) or with lymph node metastasis (42). Even though Nrf2 protein levels were increased in *KEAP1*-mutated tumors (43), current evidence does not support a prognostic role for Nrf2 or Nqo1 levels *per se*. In contrast, expression of heme oxygenase-1 (HO-1), another target gene of Nrf2 (69), was found to correlate with thyroid cancer aggressiveness (stage and risk of recurrence, plus a near-significant trend for extra-thyroidal invasion), but not with tumor size or lymph node metastasis (70). NRG1 overexpression was described as a positive prognostic factor for lymph node metastasis in PTC (58).

### Treatment

As mentioned above, Nrf2 is generally known to exert a dual role in cancer: on the one hand, it prevents cell transformation of normal cells, and on the other hand, it promotes aggressiveness, and drug resistance of malignant ones (38–41). The latter, so-called “dark side” of Nrf2 (71), has been well-studied in other cancer types, where Nrf2 has been shown to promote resistance against chemotherapy and radiotherapy (40, 41). Although better described in lung, and liver carcinomas, such drug resistance mechanisms and their implications have more recently begun to be characterized in various models of thyroid carcinoma. It is thus interesting to discuss various pharmaceutical approaches that directly or indirectly target the Nrf2 pathway as a strategy for thyroid cancer treatment.

**Figure 4:**
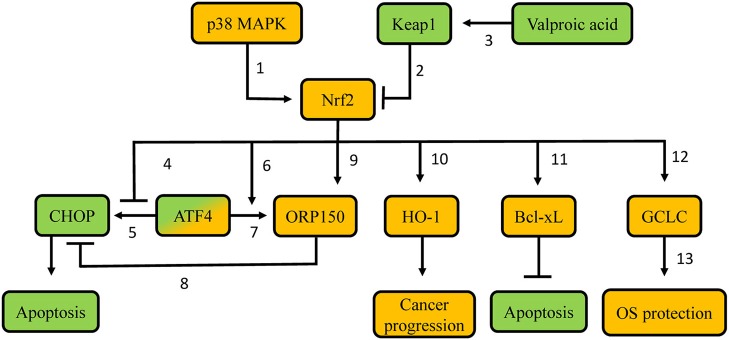
Proposed molecular mechanisms involved in resistance of thyroid carcinoma cells to proteasome inhibitors. Green-labeled molecules promote sensitivity to proteasome inhibitors, orange-labeled molecules promote resistance to proteasome inhibitors. 1. p38 MAPK phosphorylates Nrf2, thus leading to Nrf2 nuclear accumulation; 2. Keap1 targets Nrf2 for poly-ubiquitination and proteasomal degradation; 3. Valproic acid demethylates the *KEAP1* promoter, thus favoring *KEAP1* gene transcription; 4. Nrf2 precludes recruitment of ATF4 to the *CHOP* promoter, thus decreasing *CHOP* transcription; 5. ATF4 promotes *CHOP* transcription via binding to its promoter; 6. Nrf2 promotes ATF4 recruitment on the *ORP150* promoter, thus increasing *ORP150* transcription; 7. ATF4 promotes *ORP150* transcription via binding to its promoter; 8. Potential competition between ORP150 and CHOP; 9. Nrf2 promotes *ORP150* transcription; 10. Nrf2 promotes *HO-1* transcription; 11. Potentially decreased Keap1-mediated Bcl-XL poly-ubiquitination; 12. Nrf2 promotes *GCLC* transcription; 13. Gclc protein favors GSH synthesis. MAPK: Mitogen-activated protein kinase; Keap1: Kelch-like ECH-associated protein 1; Nrf2: nuclear factor erythroid 2-related transcription factor 2; CHOP: CCAAT/enhancer-binding protein homologous protein; ATF4: Activating Transcription Factor 4; ORP150: oxygen-regulated protein 150; HO-1: heme-oxygenase 1; Bcl-xL: B-cell lymphoma-extra large; GCLC: glutamate-cystein ligase, catalytic subunit; OS: oxidative stress.

Proteasome inhibitors are targeted anti-cancer agents that are in clinical use in other types of cancer. They have also been used experimentally in patients with thyroid carcinomas without established alternative treatments, such as metastatic thyroid carcinomas that are radioiodine-refractory (72). Regarding their mechanisms of action, as elucidated in other cancer types, inhibition of the proteasome leads to accumulation of misfolded proteins, inducing endoplasmic reticulum (ER) stress, and subsequent apoptosis (73). Apoptosis is induced in part via induction of CCAAT/enhancer-binding protein homologous protein (CHOP), activation of the apoptosis signal-regulating kinase 1 (ASK1)/c-Jun N-terminal kinase (JNK) pathway, and cleavage of ER-resident caspase-12 (74). Because Nrf2 is degraded in a proteasome-dependent manner, proteasome inhibition also generally leads to Nrf2 activation. Thus, a series of studies have evaluated the involvement of Nrf2 in the sensitivity of thyroid carcinoma cells lines to proteasome inhibitors. Their results and conclusions are discussed below and summarized in Figure 4.

*In vitro* experiments in poorly differentiated thyroid carcinoma (PTDC) and undifferentiated (anaplastic) thyroid carcinoma (UTC/ATC) showed that the apoptotic response to proteasome inhibitor treatment can be predicted by the induction of CHOP. Cell lines that displayed higher CHOP protein induction were more sensitive to proteasome inhibitor treatment, while experimental knock-down of CHOP partially decreased the effects of the proteasome inhibitor (75). The expression of the gene encoding CHOP is regulated by several transcription factors, including oxygen-regulated protein 150 (ORP150), an inducible ER chaperone that suppresses *CHOP* expression (76); and Activating Transcription Factor 4 (ATF4), a transcription factor induced in response to proteasome inhibition that promotes *CHOP* expression (77). Both of these transcription factors can be, at least partially, regulated by Nrf2 (78). Nrf2 increases *ORP150* gene transcription both directly, by binding to the *ORP150* gene promoter, as well as indirectly, by promoting ATF4 recruitment to the *ORP150* gene, which also upregulates its expression (78). At the same time, Nrf2 antagonizes the recruitment of ATF4 on an ARE in the *CHOP* promoter, thereby preventing ATF4-mediated *CHOP* transcription (77). Overall, Nrf2 decreases *CHOP* gene expression, hence exerting an anti-apoptotic effect in response to proteasome inhibitor treatment, and thereby favoring drug resistance. Consistent with the aforementioned mechanisms, cell lines with low sensitivity to proteasome inhibitor treatment show increased expression levels of Nrf2, and ORP150 proteins (78). Experimental knock-down of ORP150 increased the sensitivity of tumor cell lines that were less responsive to proteasome inhibitor treatment. These lines also expressed higher *CHOP* mRNA levels (76). Similarly, experimental knock-down of Nrf2 inhibited the induction of *ORP150* mRNA and protein and significantly decreased the binding of ATF4 to the *ORP150* promoter (78). These findings suggest that pharmacological inhibition of Nrf2 could be a plausible strategy to increase the sensitivity of PDTC and UTC to proteasome inhibitors.

Generation of ROS is considered to be a crucial early event (79) during the initiation of apoptosis induced by the proteasome inhibitor bortezomib in some types of cancer. Indeed, UTC cell lines that are less sensitive to bortezomib do not show an early increase in ROS (80). Additionally, those less sensitive cell lines show higher levels of GSH, which is associated with higher expression levels and activity of the Nrf2 target gene *GCLC* (80). Moreover, Nrf2 is overexpressed in these cell lines, suggesting that bortezomib resistance can be due to Nrf2-mediated synthesis of GSH (80). Other research work using UTC and PDTC models indicates that p38 MAPK, an important anti-apoptotic factor activated in response to proteasome inhibition, phosphorylates Nrf2, and promotes its accumulation in the nucleus, thereby inducing the transcription of *GCLC* (81). Pharmacological inhibition of p38 MAPK suppresses the nuclear translocation of Nrf2, thereby enhancing bortezomib-induced apoptosis, especially in bortezomib-resistant cell lines (81). These data indicate that the mechanisms by which Nrf2 promotes resistance to proteasome inhibitors in thyroid cancer are not limited to interactions with regulators of apoptosis (ATF4, ORP150, and CHOP), but also include the direct modulation of the cells' redox status.

Furthermore, overexpression of Nrf2 in PTC cells confers resistance to TRAIL (TNF-related apoptosis inducing ligand) (82). TRAIL is a molecule with growing interest in oncology, as it specifically triggers cell death (83). In TRAIL-resistant PTC, combination of the histone deacetylase inhibitor valproic acid with a TRAIL regimen leads to increased sensitivity both *in vitro*, and *in vivo* (orthotopic mouse model of PTC) (82). Interestingly, in a different epithelial cell model (human lens), valproic acid is also known to promote demethylation of the *KEAP1* promoter, leading to upregulation of *KEAP1* expression, and subsequent decreased Nrf2 protein abundance (84). Consistent with this, in TRAIL-resistant PTC, combination therapy with valproic acid and TRAIL decreases the nuclear levels, and activity of Nrf2, leading to downregulation of Bcl-xL, an anti-apoptotic molecule of the Bcl-2 family (85). Experimental knock-down of Nrf2 also decreases Bcl-xL protein levels, thereby promoting apoptosis in cancer cells (82). Interestingly, Keap1 has been shown to negatively regulate the activity of Bcl-xL by targeting it for poly-ubiquitination (86). These data indicate that Nrf2 promotes resistance not only to proteasome inhibitors, but also to other experimental therapies, and it could thus be considered as a candidate target to increase sensitivity to such treatments when they are tested in clinic trials. However, although this approach to treat/prevent PTC appears mechanistically appealing, it may have unexpected adverse effects. Indeed, human lens epithelial cells treated with 5-aza-2′deoxycytidine (a compound known to promote demethylation of CpG islands in the *KEAP1* promoter) displayed increased Keap1 protein levels, decreased Nrf2 stabilization, enhanced ROS, and increased cell death. These alterations resulted in a diabetic cataract lens phenotype (87), which is consistent with the fact that demethylation of the *KEAP1* promoter is also associated with enhanced age-related cataract in humans (88).

Finally, inhibitors of HO-1 have been shown to decrease proliferation, migration, and invasion of follicular thyroid carcinoma (FTC) and UTC cell lines, and to reduce FTC tumor growth in a xenograft model (89). This study demonstrates that desirable effects on thyroid carcinoma might be achieved not only by targeting Nrf2 directly, but also potentially by targeting other components of the Keap1/Nrf2 pathway and its downstream signaling.

## Conclusion

In summary, Keap1/Nrf2 signaling is involved in both benign and malignant thyroid conditions, where it might serve as a prognosis marker, or therapeutic target. Ongoing research, including cell culture studies with normal and transformed thyroid follicular cells, thyroidal phenotyping of animal models, analysis of human thyroid tissue samples, and monitoring of thyroid function and volume in clinical studies, is expected to yield a better understanding of the involvement of Nrf2 in thyroid physiology, and pathophysiology. Such work is important in order to ensure the thyroidal safety of Nrf2-activating compounds that are being developed for use in other indications, and it might also facilitate the development of new drugs for the prevention and the treatment of both benign thyroid diseases, and thyroid carcinomas.

## Author Contributions

CR drafted and edited the manuscript and the figures. PZ and MB edited the manuscript. DC prepared figures, edited the manuscript, and contributed to the discussion. GS conceived and edited the manuscript.

### Conflict of Interest Statement

The authors declare that the research was conducted in the absence of any commercial or financial relationships that could be construed as a potential conflict of interest.
